# Ultrahigh-*Q* integrated flame-hydrolysis-deposited germano-silicate resonators on silicon

**DOI:** 10.1038/s41377-026-02353-y

**Published:** 2026-06-04

**Authors:** Hao-Jing Chen, Kellan Colburn, Hanfei Hou, Hongrui Yan, Avani Ranka, Jin-Yu Liu, Lue Wu, Bruno Moog, Oleksandr Buchnev, Stefano Fornetti, Christopher Holmes, James Gates, Dirk Bouwmeester, Henry Blauvelt, Kerry Vahala

**Affiliations:** 1 T. J. Watson Laboratory of Applied Physics, California Institute of Technology, Pasadena, CA USA; 2 Optoelectronics Research Centre, University of Southampton, Southampton, UK; 3 School of Engineering, University of Southampton, Southampton, UK; 4 Department of Physics, University of California Santa Barbara, Santa Barbara, CA USA; 5 Huygens-Kamerlingh Onnes Laboratory, Leiden University, Leiden, The Netherlands

**Keywords:** Silicon photonics, Integrated optics

## Abstract

Optical fibres, owing to their ultra-low transmission loss, underpin global telecommunications. However, this remarkable low-loss performance has not been extended to integrated photonic devices, which are increasingly critical for data-intensive communications in the era of artificial intelligence (AI). Here, we translate the widely adopted mass-production process for fibre manufacturing—flame hydrolysis—to wafer-scale integrated photonics, and demonstrate ultrahigh-*Q* integrated microresonators. By leveraging high GeO_2_ doping, the deposited germano-silicate (Ge:silica) films achieve full densification at moderate thermal budgets, while also allowing for a post-processing furnace-reflow technique that has the capability to both repair any etch-induced defects and enhance optical *Q*, leading to a high degree of process tolerance. When combined with deep-UV lithography, these films form microresonators exhibiting ultrahigh *Q* factors of up to 566 million at 1064 nm, corresponding to a waveguide propagation loss as low as 0.07 dB m^−1^. Like their fibre counterparts, these devices exhibit a broad transmission window with *Q* factors surpassing 100 million demonstrated from the telecommunications band to the violet spectrum. Moreover, the width dependence of *Q* factor and a two-order-of-magnitude *Q* recovery enabled by the furnace reflow process are also demonstrated. This work extends high-quality, high-rate flame hydrolysis deposition (FHD) from optical fibre manufacturing to integrated photonics, establishing a scalable route towards fibre-level loss in photonic integrated circuits.

## Introduction

Photonic integrated circuits (PICs) have emerged as transformative platforms across science and technology, enabling compact, scalable solutions for optical communications^1,2^, quantum computing^3,4^, biosensing^5^ and precision metrology^6,7^. At the heart of PIC performance lies a critical parameter, waveguide propagation loss. For instance, low loss preserves optical signal integrity for communications and minimizes photon loss in quantum systems, ensuring high-fidelity entanglement^8,9^. Critically, low-loss waveguides enable high-*Q* resonators, which dramatically reduce power requirements^10,11^ and enhance the laser coherence of on-chip devices^12,13^, making it possible for system level integration with high performance^14–16^. The past decade has witnessed landmark advances in the fabrication of certain integrated photonic platforms, most notably silicon nitride (Si_3_N_4_)^17,18^ and lithium niobate (LiNbO_3_)^19^. Si_3_N_4_ platforms, with losses below 1 dB m^−1^ at telecom wavelengths, have enabled chip-based optical clocks^20^ and petabit-scale communications^21^. Thin film LiNbO_3_ and LiTaO_3_, leveraging their strong Pockels effect and nonlinearity, have facilitated high-speed electro-optic modulation^22,23^ and high-efficiency frequency conversion^24^. However, these platforms are already approaching their intrinsic material absorption limits^25^, leaving little room for further improvement, and their losses remain more than 20 dB higher than those of standard telecom optical fibre (Ge:silica core with silica cladding).

In the last century, Corning produced the first practical low-loss optical fibre by utilizing flame hydrolysis for high-purity doped-silica glass^26^. This technique relies on gaseous precursors, thereby eliminating contamination from metallic impurities that plagued earlier methods. Owing to its rapid deposition rate and cost-effectiveness, flame hydrolysis soon inspired derivative processes such as outside vapor deposition (OVD) and vapor axial deposition (VAD), which remain the foundation of modern optical fibre manufacturing to this day^27^. Flame hydrolysis has also been adapted for the fabrication of Ge:silica planar lightwave circuits^28–31^. Devices such as arrayed waveguide gratings (AWGs) and Bragg gratings have been widely deployed in optical communications^32^, while Mach-Zehnder interferometers (MZIs) have served as pioneering platforms in quantum information processing^33^. More recently, significant reductions in propagation loss have been achieved in Ge:silica integrated photonics ( ~ 25 mol% GeO_2_ doping via plasma-enhanced chemical vapor deposition (PECVD))^34^, demonstrating that this material system can approach the ultralow-loss regime required for high-coherence applications.

Here, by systematically optimizing flame hydrolysis wafer deposition, waveguide fabrication, and post-etch thermal annealing, we demonstrate ultrahigh-*Q* integrated microresonators based on high-GeO_2_-doped silica ( ~ 50 mol%), spanning from the telecom band to the violet spectrum. This expanded high-doping compositional window lowers the glass viscosity, reducing the required thermal reflow budget, while the increased refractive index contrast enables smaller achievable bending radii. The record-high *Q* factor of 566 million is achieved at 1064 nm, while *Q* factors exceeding 100 million are demonstrated across a broad spectral range from 458 nm to 1550 nm. This sub-0.1 dB m^−1^ loss performance represents a critical step toward realizing fibre-like loss on a chip, with the potential to dramatically enhance the performance of integrated photonics for broadband lasers^35^ and frequency combs^36^, along with applications in cold atom physics, optical communication and quantum computing circuits.

## Results

**Fig. 1:**
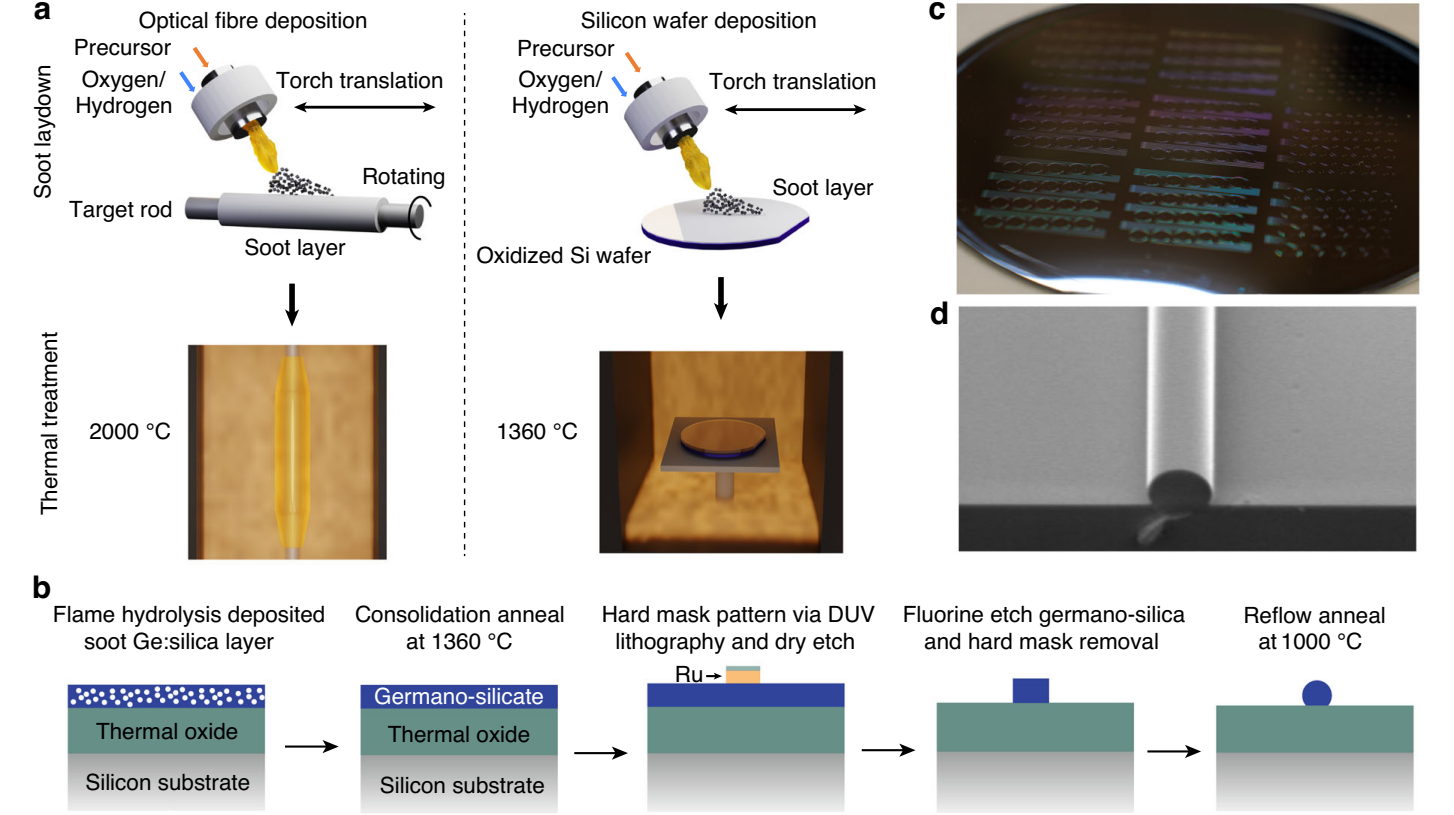
Flame-hydrolysis-deposited optical-fibre-like circuits on silicon wafer. **a** Schematic of Ge:silica layer deposition on fibre preform and silicon wafer. In conventional fibre manufacturing, silica soot is deposited by high-temperature flame hydrolysis of precursors onto a rotating rod, followed by consolidation and drawing at up to 2000 °C to form the preform. In this work, we adapt the process to deposit onto oxidized silicon wafers mounted on a rotating stage. Owing to the higher GeO_2_ doping level, the deposited films exhibit a reduced glass transition temperature, allowing full consolidation at 1360 °C. **b** Workflow of waveguide fabrication process. **c** Photograph of Ge:silica wafer containing devices. **d** Cross-section scanning electron microscope (SEM) image of a reflowed waveguide

Figure 1 a illustrates the use of flame hydrolysis in both optical fibre manufacturing (OVD is used as an example) and planar lightwave circuits. In this process, chloride-based precursors (e.g., SiCl_4_, GeCl_4_) undergo direct oxidation and hydrolysis in an oxygen-hydrogen flame. Dopants such as germanium, boron, and phosphorus are incorporated to tailor the photosensitivity, refractive index, and internal stress of the deposited material. In fibre manufacturing, flame hydrolysis generates a porous white soot layer on a continuously rotated quartz silica starting rod. This soot layer is subsequently consolidated at temperatures approaching 2000 °C into a transparent and homogeneous glass preform, which is then drawn into optical fibres. In planar lightwave circuits, however, the soot is deposited onto oxidized silicon wafers mounted on a rotating stage, enabling uniform thin-film coverage. In this work, the GeO_2_ doping concentration is ~ 50 mol%, significantly higher than the ~ 3.5 mol% typically employed in optical fibres. Such heavy doping not only provides a high refractive index contrast of Δ*n* ≈ 4% relative to pure silica, but also lowers the material’s softening and melting temperature. The deposited wafers are subsequently consolidated in a vertical furnace at 1360 °C, a reduced thermal budget compared with fibre preform processing. This temperature is carefully chosen to achieve full densification of the material while mitigating silicon substrate roughening and stress at the silica-silicon interface.

The detailed waveguide fabrication workflow is shown in Fig. 1b. In the devices studied, Ge:silica films with a thickness of ~ 10 μm were deposited via FHD on top of an 8–15 μm-thick thermally grown SiO_2_ layer on silicon (see detailed deposition process in Methods). The FHD layers were then patterned into ridge waveguides using deep-ultraviolet (DUV) lithography and dry etching. For lithographic processing, the wafer was first coated with a hard mask comprising a 100–300 nm ruthenium film (sputtered)^37^ and a 10 nm silica adhesion layer (deposited by atomic layer deposition, ALD). The high selectivity of the Ru mask in fluorine-based chemistries enables precise profile control and high-fidelity deep etching of Ge:silica. A bottom anti-reflective coating (BARC) and photoresist were then spun on and patterned using DUV lithography. The resulting three-layer mask design was subsequently transferred into the germano-silica by inductively coupled plasma (ICP) etching: fluorine-based etching was used to open the ALD silica, oxygen-chlorine etching was used for ruthenium removal, and fluorine-based etching was again applied to define the germano-silica waveguides. From there, the remaining hard mask was removed by ICP etching, leaving only the desired waveguide structures. Finally, to mitigate sidewall roughness and repair any etch-induced defects, the entire wafer was subjected to thermal reflow in a high temperature furnace. By tuning the annealing temperature (viscosity of the glass) and time, the waveguide cross-section could be reshaped: at sufficiently high temperatures surface tension becomes high enough to fully transform the ridges into nearly circular profiles, resembling optical fibres on a chip. The whole fabrication process is carried out at the wafer scale and is compatible with large-scale production of ultra-low-loss photonic integrated circuits. A photograph of a processed wafer with patterned devices, along with a scanning electron microscope (SEM) image of a thermally reflowed, rounded waveguide cross-section, are shown in Fig. 1c,d.

**Fig. 2:**
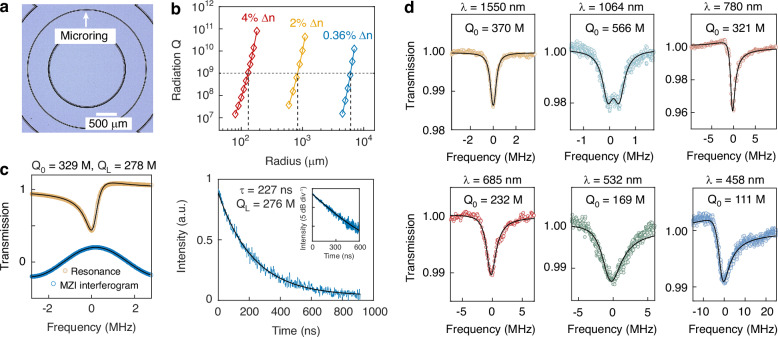
Characterization of Ge:silica integrated microresonators. **a** Optical micrograph of a Ge:silica microring resonator. **b** Simulated radiation *Q* factors at 1550 nm for resonators with different Ge doping levels. The bending radii corresponding to 4% Δ*n* (red trace, this work), 2% Δ*n* (yellow trace, ref. ^34^), and 0.36% Δ*n* (blue trace, standard SMF-28 fibre) are 130 μm, 840 μm, and 6 mm, respectively. The bending radius is defined as the value at which the radiation *Q* reaches 10^9^. **c** *Q* measurement at 1550 nm using both MZI interferogram and cavity ringdown methods, yielding loaded *Q* factors of 278 M and 276 M, respectively. Inset: log scale of the ringdown trace. **d** Broadband intrinsic *Q* measurements under weak-coupling conditions, achieving ultrahigh *Q* factors of 370 M at 1550 nm, 566 M at 1064 nm, 321 M at 780 nm, 232 M at 685 nm, 169 M at 532 nm, and 111 M at 458 nm. The observed double-dip features at 1064 nm arise from mode splitting induced by backscattering in the microcavity^48^, whereas the Fano lineshapes at 780 nm, 532 nm and 458 nm originate from multimode interference^49^ (see detailed analysis in Methods)

To characterize the ultralow waveguide loss, microring resonators are fabricated (Fig. 2a), whose *Q* factors provide a highly sensitive indication of propagation loss. To ensure that bending loss does not impose a limitation, the radiation *Q* at 1550 nm was simulated (details see Methods), with the results plotted in Fig. 2b. Owing to the relatively high index contrast of Δ*n* ≈ 4% employed here, the bending radius can be reduced to 130 μm, defined as the radius at which the radiation *Q* reaches 10^9^. For comparison, devices with Δ*n* = 2% and Δ*n* = 0.36% (the typical fibre value) require bending radii of ~ 840 *μ*m and 6 mm, respectively. Based on these considerations, the devices studied here were designed with microring diameters of 1.5 mm. To investigate the dependence of the *Q* factor on waveguide width, the microring width was varied from 3 to 30 μm, while the waveguide thickness was fixed at approximately 10 *μ*m. For rapid and flexible testing over a broadband wavelength range, a U-shaped fibre taper was employed to couple light into the resonator. The *Q* factors were characterized by recording transmission spectra using a tunable external-cavity laser, calibrated against a Mach-Zehnder interferometer. From the measured resonance linewidths, the loaded, coupling, and intrinsic *Q* factors were extracted. Fig. 2c illustrates a typical measurement at 1550 nm. Due to multimode interference, the resonance spectrum exhibited a Fano-like lineshape (Fig. 2c, left). By applying a corrected Lorentzian fitting procedure (see Methods), we obtained an intrinsic *Q* of 329 million and a loaded *Q* of 278 million. To independently verify these results, cavity ring-down measurements were performed on the same resonator. An intensity modulator was inserted between the laser and cavity to serve as a fast optical switch. The resulting decay trace (Fig. 2c, right) shows a photon lifetime of 227 ns, corresponding to a loaded *Q* of 276 million, in full agreement with the transmission-based measurement.

The coupling was then adjusted to a weakly coupled regime to minimize coupler-induced loss and obtain the optimal intrinsic *Q*. Measurements were performed from 1550 nm down to 458 nm. As shown in Fig. 2d, the highest *Q* of 566 million was observed at 1064 nm, benefiting from a balance between OH absorption at longer wavelengths and scattering losses at shorter wavelengths. The resonance spectrum appears as doublets, a consequence of the ultrahigh *Q* combined with backscattering within the waveguide. Thanks to the complete thermal reflow process, surface tension smoothed the sidewalls and substantially reduced scattering, enabling the maintenance of *Q* factors above 100 million down to 458 nm. The slight decrease in *Q* at shorter wavelengths is likely attributable to absorption associated with the high GeO_2_ doping, which has known absorption peaks in the ultraviolet band^38,39^. This level of visible-band performance surpasses other integrated photonic platforms by more than an order of magnitude, and holds significant promise for applications in optical clocks, quantum control, astronomical observation, and bioimaging.

**Fig. 3:**
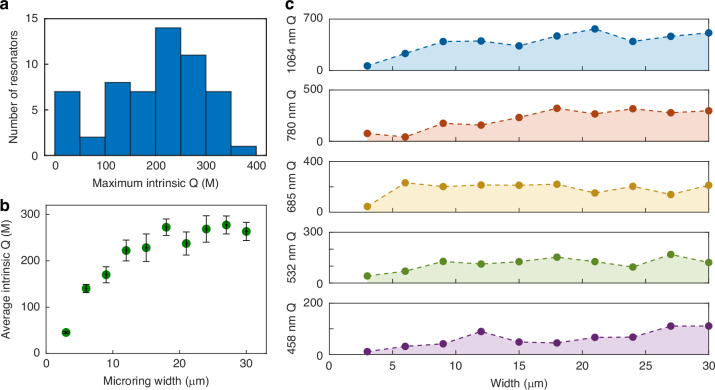
Statistics of *Q* factors and their dependence on waveguide width. **a** Distribution of *Q* factors measured at 1550 nm from 60 resonators with varying widths. Resonators with *Q* < 50 million are associated with the narrowest widths. **b** Average intrinsic *Q* as a function of microring width at 1550 nm. **c** Maximum intrinsic *Q* as a function of microring width at 1064, 780, 685, 532 and 458 nm. At each wavelength, an array of 10 microrings was used as the sample

Next, we investigated the wafer-scale fabrication yield. Six arrays of microrings, each with widths ranging from 3 to 30 μm and diameters of 3 mm, were selected from different regions of the wafer, yielding a total of 60 resonators. Fig. 3a presents a histogram of the maximum intrinsic *Q* at 1550 nm for each device. Apart from the narrowest rings (3 and 6 μm), which contributed six resonators with *Q* < 50 million, the remaining devices exhibited *Q* values centered around 200-250 million, with a distribution spanning 50–400 million. These results highlight the high uniformity and reproducibility of wafer-scale fabrication. The dependence of the average intrinsic *Q* on microring width at 1550 nm is shown in Fig. 3b. The *Q* factor increases with width and saturates beyond ~ 12 μm. Figure 3c presents the corresponding results across other wavelengths (458-1064 nm). Apart from a few cases exhibiting anomalously high *Q* values (likely arising from higher-order modes), the overall trend is consistent with that observed at 1550 nm. These results suggest that additional loss mechanisms, such as water-vapor absorption at the waveguide-air interface, may be present. Increasing the waveguide width confines the optical mode more effectively within the Ge:silica core, thereby reducing the fraction of the optical field at the interface and improving the *Q* factor.

**Fig. 4:**
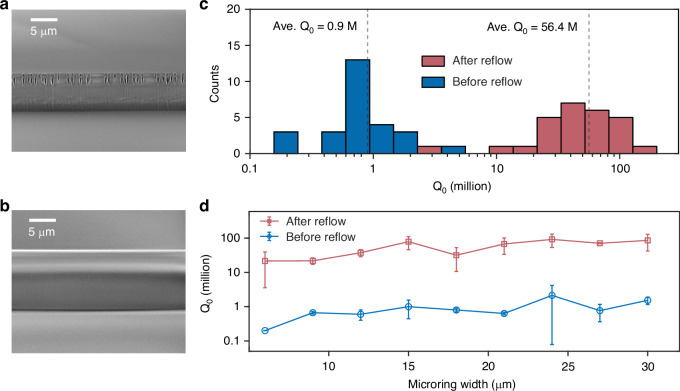
Sidewall repair using a furnace reflow process. **a**, **b** SEM images of the waveguide sidewall before (**a**) and after (**b**) the furnace reflow process. The reflowed waveguide becomes noticeably rounded, which also leads to charging effects in SEM image. **c** Statistical distribution of the resonator *Q* before and after reflow, showing an average enhancement from 0.9 M to 56.4 M. **d** Comparison of the resonator *Q* as a function of waveguide width before and after reflow

In addition, the furnace reflow process provides substantial tolerance against etch imperfections. Even when the initial etch quality is poor and the resonator exhibits an intrinsic *Q* of only ~ 1 M or lower, the reflow step can significantly reduce the sidewall roughness, enabling ultrahigh-*Q* values of up to 100 M. Fig. 4a shows the sidewall SEM image of an intentionally poorly etched waveguide (bias current on ICP was intentionally set too high to induce channeling and sputter re-deposition). After an 18-hour furnace reflow at 1000 °C, the sidewall becomes significantly smoother (Fig. 4b). Surface tension during reflow also rounds the originally rectangular waveguide cross-section (see the detailed quantitative geometric analysis in the Supplementary Information), which introduces noticeable charging effects in the SEM. It is noted that Fig. 4a corresponds to a wide waveguide, where only one sidewall is visible in the SEM, whereas Fig. 4b corresponds to a narrow waveguide, allowing the full profile to be observed. To systematically evaluate the etch repair ability, we examined 27 resonators at a wavelength of 1550 nm, with widths ranging from 6–30 *μ*m across three different chips. Figure 4c shows the statistical distributions of the *Q* values before and after reflow. The average *Q* improves dramatically from 0.9 M to 56.4 M, representing nearly two orders of magnitude enhancement. The highest measured *Q* reaches 132 M, compared to only 1.1 M before reflow. Figure 4d summarizes the *Q* improvement as a function of waveguide width, showing that all devices experience substantial enhancement after reflow.

## Discussion

In summary, we have demonstrated an integrated photonics platform for fabricating Ge:silica integrated resonators that achieve ultra-high *Q* factors across a broad spectral range. This work shows that flame hydrolysis deposition in optical fibre manufacturing permits high-quality thin-film wafer deposition for integrated photonics. The high concentration of GeO_2_ doping significantly reduces the required bending radius and lowers the consolidation and reflow temperatures. The unique reflow characteristics provide enhanced tolerance to etching variations while preserving ultralow optical loss across a broad wavelength range. When combined with advanced coupling designs^40^ and integrated amplification^41^, this platform paves the way for high-performance broadband applications, including self-referenced frequency combs and multi-wavelength light sources for atomic and ionic systems.

Compared to higher-density or strongly nonlinear platforms^42–45^, the Ge:silica platform is distinguished by its ultrahigh *Q* factor (particularly at shorter wavelengths) and in large-mode-area geometries, enabling high coherence, low power operation, reduced thermorefractive noise, and enhanced power handling. Such performance is critical for applications including optical clocks, quantum photonics, coherent communications, and precision sensing. Looking forward, further improvements may be achieved through precise reflow engineering for optimized resonator morphology, reduction of OH absorption via dehydration treatments^46^, and incorporation of active dopants to realize on-chip lasers and amplifiers. The ability to combine wafer-scale manufacturability with fibre-level optical performance opens the door to a new generation of photonic integrated circuits spanning from classical to quantum technologies.

## Methods

### Flame hydrolysis deposition

Flame hydrolysis deposition (FHD) was conducted on 100 mm diameter silicon wafers with an 8 μm thermally grown wet oxide. The FHD system was configured in a downward orientation, with wafers placed upright upon a spinning silicon carbide turntable heated to 200 °C, and the FHD torch angled downward at 30° relative to the plane normal of the turntable. The FHD torch consists of concentric fused-silica annular flow channels that deliver chloride-based precursors, hydrogen, oxygen, and an argon sheath to shape the flame. To form the soot, the torch was supplied with H_2_ and O_2_ at 5 L ${\min }^{-1}$. The flame was shaped by an Ar flow of 8 L ${\min }^{-1}$ and co-delivery of silicon tetrachloride (SiCl_4_) and germanium tetrachloride (GeCl_4_) using a bubbler system (SG Controls) at flow rates of 30 sccm and 190 sccm, respectively. Uniform deposition on the wafer was achieved by translating the torch radially across the rotating turntable. The maximum surface speed was set to 628 mm s^−1^, with the table drive gear ratio corresponding to 2030 μm of radial travel per revolution. The table rotation speed was actively controlled during the radial traverse of the torch to maintain a constant deposition per unit area across the turntable. Deposition was carried out at 1015 mbar with a total of 14 deposition passes, where a single pass relates to either a positive or negative radial translation. Following deposition, films were consolidated by heating in flowing oxygen (0.9 L $\min^{-1}$) at a rate of 5 K $\min^{-1}$ up to 1360 °C, where they were held for 2 h. The refractive index and thickness of the resulting film were subsequently measured using a Metricon prism coupler, yielding *n* = 1.5149 at a wavelength of 1550 nm.

### Simulation of radiation loss and bending radius

Radiation *Q* is defined as the quality factor limited by the energy loss due to coupling into the silicon substrate and bending loss from the curvature of the resonator. This is modeled through 2D-finite-element eigenmode simulations using the Electromagnetic Waves, Frequency Domain (EWFD) module in COMSOL Multiphysics^®^. Perfectly matched layers (PML) are introduced along the boundary of the simulation region to absorb and capture this loss. The waveguide geometry is chosen to be representative of our platform: a 12-*μ*m-wide, 10-*μ*m-high Ge:silica rectangular core positioned on top of a 15 μm-thick thermally grown silica undercladding, with no upper cladding. Waveguide simulations are performed at 2%Δ*n* and 4%Δ*n* index contrast between core and bottom cladding. For the SMF-28 fibre simulation, we use the nominal geometry and refractive indices provided by Corning^®^. The fibre has an 8.2 μm-diameter core with an index of 1.4492, surrounded by a cladding of diameter 125 μm and index 1.4440, corresponding to an index contrast of 0.36%. Numerically, the FEM simulation will return a complex eigen index of the mode, whose real part encapsulates the resonant frequency and imaginary part encapsulates the loss. Radiation *Q*, then by definition, is calculated using the real (Re(*n*_eff_)) and imaginary (Im(*n*_eff_)) parts of the effective mode index^47^:

1 $${Q}_{{\rm{rad}}}=\frac{Re({n}_{{\rm{eff}}})}{2\cdot Im({n}_{{\rm{eff}}})}$$

### Resonance lineshape fitting

The resonance spectra in Fig. 2 were analyzed using standard temporal coupled-mode theory and a fitting protocol similar to that used in previous work^34^, with Lorentzian, mode-splitting, and Fano-type lineshapes applied depending on the measured resonance profile. The Lorentzian transmission fitting function is given by

2 $$T(\omega )=1-\frac{{\kappa }_{{\rm{ext}}}{\kappa }_{0}}{{({\omega }_{0}-\omega )}^{2}+{(\kappa /2)}^{2}}$$

where *ω*_0_ is the cavity resonance frequency, *κ*_0_ is the intrinsic loss rate, *κ*_ext_ is the external coupling rate, and *κ* = *κ*_0_ + *κ*_ext_ is the total loss rate. From this fit, both *κ*_ext_ and *κ*_0_ can be extracted, yielding the intrinsic quality factor *Q*_0_ = *ω*_0_/*κ*_0_.

In the presence of coherent backscattering or coupling between counter-propagating modes, the originally degenerate resonance splits into a symmetric and an antisymmetric mode. The transmission spectrum therefore exhibits a characteristic mode-splitting-induced doublet. In this case, the transmission function is modified to

3 $${T}_{{\rm{split}}}({\omega }_{p})\simeq {\left|1-\frac{{\kappa }_{{\rm{ext}}}}{i(\Delta -g)+\kappa /2}-\frac{{\kappa }_{{\rm{ext}}}}{i(\Delta +g)+\kappa /2}\right|}^{2}$$

which corresponds to the widely adopted mode-splitting doublet model. Fitting this expression yields not only the intrinsic loss rate *κ*_0_ and external coupling rate *κ*_ext_, but also the mode-splitting strength 2*g*, from which the intrinsic quality factor can again be extracted as *Q*_0_ = *ω*_0_/*κ*_0_.

When an interfering pathway, such as multimode interference in the coupler or the presence of a continuum-like low-*Q* background mode, couples to the primary high-*Q* resonance, the transmission lineshape becomes asymmetric and exhibits a Fano resonance. The transmission function is modified to

4 $${T}_{{\rm{Fano}}}=\frac{{\left[q+2({\omega }_{0}-{\omega }_{p})/\kappa \right]}^{2}}{1+{\left[2({\omega }_{0}-{\omega }_{p})/\kappa \right]}^{2}}$$

where *q* is the Fano parameter describing the degree of asymmetry. Fitting the spectrum with Eq. (4) yields *κ*_0_, *κ*_ext_, and *q*, thereby enabling extraction of the intrinsic quality factor *Q*_0_ = *ω*_0_/*κ*_0_ and quantification of interference-induced distortions in the resonance lineshape.

## Supplementary information

Supplementary information for Ultrahigh-4; integrated flame-hydrolysis-deposited germano-silicate resonators on silicon

## Data Availability

The data that support the plots within this paper are available in Zenodo at 10.5281/zenodo.20373763. Additional data are available from the corresponding author upon reasonable request.
