# Supplementary material for: Ultrahigh-Q integrated flame-hydrolysis-deposited germano-silicate resonators on silicon
Source: Light Sci Appl. 2026 Jun 4;15:265. doi: 10.1038/s41377-026-02353-y (PMC13234339; doi:10.1038/s41377-026-02353-y)
Supplement: Supplementary file 1 — Supplementary information for Ultrahigh-4; integrated flame-hydrolysis-deposited germano-silicate resonators on silicon [file 41377_2026_2353_MOESM1_ESM.pdf]

Supplementary Information for

Ultrahigh-Q integrated flame-hydrolysis-deposited germano-silicate resonators on silicon

Hao-Jing Chen[1,*,†], Kellan Colburn[1,*,†], Hanfei Hou[1], Hongrui Yan[1], Avani Ranka[1], Jin-Yu Liu[1], Lue Wu[1], Bruno Moog[2], Oleksandr Buchnev[2], Stefano Fornetti[2], Christopher Holmes[2,3], James Gates[2], Dirk Bouwmeester[4,5], Henry Blauvelt[1] and Kerry Vahala[1,†]

[1]T. J. Watson Laboratory of Applied Physics, California Institute of Technology, Pasadena, CA, USA.

[2]Optoelectronics Research Centre, University of Southampton, Southampton, UK.

[3]School of Engineering, University of Southampton, Southampton, UK.

[4]Department of Physics, University of California Santa Barbara, Santa Barbara, CA, USA.

[5]Huygens-Kamerlingh Onnes Laboratory, Leiden University, Leiden, The Netherlands.

[*]These authors contributed equally to this work.

[†]e-mail: haojing@caltech.edu, kcolburn@caltech.edu, vahala@caltech.edu

Contents

1 Loss mechanism discussion **2**

1.1 Scattering loss 2

1.2 Absorption loss 3

1.3 Radiation loss 5

2 Details on device characterization and design **6**

2.1 Thickness and refractive index uniformity across the wafer 6

2.2 Run-to-run stability and long-term process reproducibility 6

2.3 Rationale for the GeO_2 doping concentration 7

2.4 Waveguide morphology evolution during thermal reflow 8

3 Comparison with Ge:silica and other integrated photonic platforms **9**

3.1 Comparison of Ge:silica integrated photonic platforms 9

3.2 Comparison with other ultralow-loss integrated photonic platforms 10

1 Loss mechanism discussion

In this section, we analyze and discuss the dominant optical loss mechanisms in the ultrahigh-Q microresonators presented in this work. The total propagation loss can be decomposed into absorption loss, scattering loss, and radiation loss. By combining wavelength-dependent measurements, geometric scaling analysis, numerical simulations, and material characterization, absorption (particularly surface OH/water-related absorption) appears to be the dominant loss channel after furnace reflow, while scattering and radiation losses are likely subdominant.

1.1 Scattering loss

Scattering loss arises from surface and sidewall roughness, as well as from bulk inhomogeneities in the refractive index. In the present devices, surface and sidewall scattering are strongly suppressed by furnace reflow. Atomic force microscopy (AFM) measurements show a post-reflow surface roughness of $\sim 0.2\,\text{nm}$ RMS and correlation length $L_c \approx 20\,\text{nm}$ (Fig. S1). Owing to the ultralow surface roughness achieved after thermal reflow and the large whispering–gallery round–trip scale ($D = 3$ mm), the surface–scattering loss can be estimated using the Rayleigh–type surface–scattering limit for whispering–gallery resonators,

$$Q_{\text{sca}} = \frac{\lambda^2 D}{2\pi^2 \sigma^2 L}, \tag{1}$$

as derived by Gorodetsky *et al.* [Opt. Lett. 21, 453 (1996)]. Using this expression, the estimated scattering–limited quality factor is $Q_{\text{sca}} \approx 5 \times 10^{10}$ at $\lambda = 458$ nm and $Q_{\text{sca}} \approx 6 \times 10^{11}$ at $\lambda = 1550$ nm. These values are more than two orders of magnitude higher than the experimentally measured intrinsic quality factors (e.g., $Q_{\text{meas}} \sim 1.1 \times 10^8$ at 458 nm and $\sim 3.7 \times 10^8$ at 1550 nm). This clear separation demonstrates that, after reflow, surface–scattering loss is not the dominant loss mechanism across the entire 450–1550 nm wavelength range.

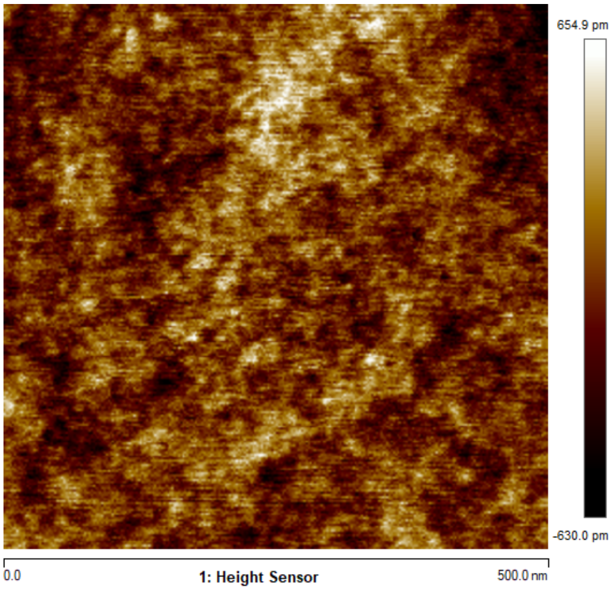

Fig. S1: AFM characterization of the reflowed Ge:silica device surface shows a Gaussian-correlated roughness with RMS roughness $\sigma \approx 0.18\,\mathrm{nm}$ and correlation length $L_c \approx 20\,\mathrm{nm}$, obtained from corrected ACF and PSDF analyses.

1.2 Absorption loss

Once scattering is mitigated by thermal reflow, absorption becomes the dominant loss mechanism. We identify three primary absorption contributions: bulk absorption associated with Ge doping, OH-related absorption, and contamination-related absorption.

(i) **Bulk absorption associated with GeO_2 doping**, which primarily affects the near-UV and visible wavelengths. As reported previously, Ge-doped silica exhibits increased absorption toward shorter wavelengths [Applied Optics 21, 136–140 (1982), Applied Optics 36, 6809–6814 (1997)]. Consistent with this behavior, Fig. S2 shows that the present devices exhibit higher intrinsic Q in the infrared compared to lower-doped Ge:silica resonators reported in [Nature 649, 338–344 (2026)], while exhibiting reduced Q in the violet and green spectral regions. This trend provides direct experimental evidence that the higher Ge doping level predominantly introduces additional loss in the short-wavelength regime.

(ii) OH-related absorption. OH-related absorption is a well-known historical limitation for optical-fibre loss in the infrared. In our devices, although high-temperature annealing significantly reduces the OH content in the bulk Ge:silica, a residual OH concentration is expected to remain. In

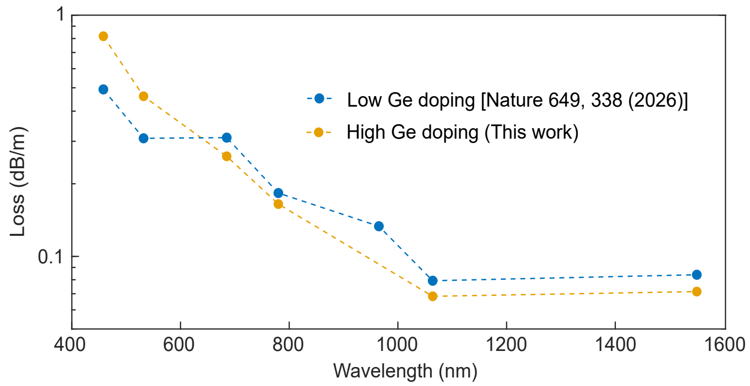

Fig. S2: **Loss comparison between high-GeO_2-doped and low-GeO_2-doped Ge:silica waveguides.** The high-GeO_2-doped Ge:silica devices developed in this work exhibit lower propagation loss in the near-infrared compared with the low-GeO_2-doped Ge:silica devices reported in *Nature*. At shorter wavelengths, however, increased GeO_2-induced absorption leads to higher loss, causing the propagation loss to surpass that of the low-GeO_2 devices.

addition, because the devices do not employ an upper cladding, the exposed silica surface can form new Si–OH bonds and a thin adsorbed water layer when operated in air. The combined absorption from residual bulk OH and surface-bound OH/water therefore likely constitutes the dominant loss mechanism that currently limits the measured quality factors. Importantly, according to the detailed analysis of OH vibrational overtones and combination bands in synthetic silica by [Humbach *et al.*, J. Non-Cryst. Solids (1996)], the dominant OH absorption bands are located near 1.39 μm and 1.24 μm, while higher-order overtones below 1 μm are significantly weaker. As a result, 1064 nm lies in a spectral minimum between the main OH absorption bands, where only weak tail absorption remains. This explains the existence of a low-OH-absorption window near 1064 nm and is fully consistent with our experimental observation that the highest intrinsic quality factor is obtained at 1064 nm. To further assess the impact of surface-bound water on the optical loss, we performed numerical simulations evaluating the overlap between the optical mode and a thin surface water layer. The simulations show that as the waveguide width increases, the optical field overlap with the surface water layer is reduced, leading to a corresponding increase in the simulated quality factor. Beyond a certain waveguide width, the quality factor saturates as the surface overlap becomes negligible. The simulated trend, shown in Fig. S3, is in good quantitative agreement with the experimentally observed dependence of Q on waveguide width, providing strong evidence that

surface OH/water absorption is a major contributor to the current loss budget.

(iii) **Contamination–related absorption.** Trace contaminants introduced during fabrication can induce additional optical loss, particularly at the ultrahigh-Q level. This effect is more pronounced in a university cleanroom environment than in a dedicated optical-fibre or photonics foundry, where contamination control and process integration are typically more tightly optimized. While contamination-related absorption is inherently difficult to quantify with high precision, it mainly affects device yield rather than the intrinsic performance metrics of interest here. Accordingly, our analysis focuses on the best-performing devices with negligible contamination, and contamination-related absorption is not quantitatively discussed.

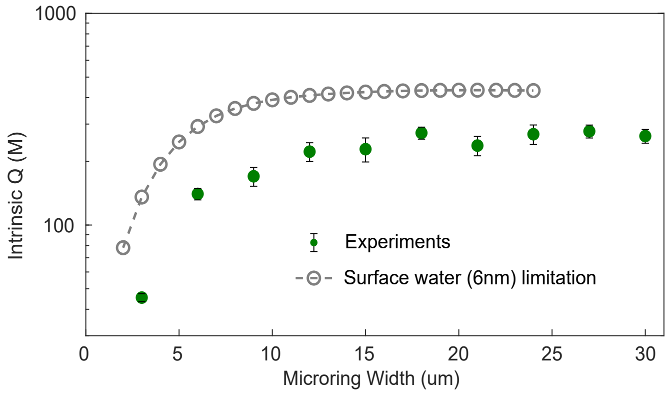

Fig. S3: **Quality factor versus waveguide width at 1550 nm.** The simulated water-absorption-limited quality factor exhibits a similar dependence on waveguide width as the experimentally measured Q, indicating that surface water absorption is likely a dominant contributor to the observed optical loss.

1.3 Radiation loss

Radiation loss consists of bending-induced radiation and substrate leakage. When the resonator curvature radius is too small, the centrifugal potential experienced by the circulating mode leads to radiation into the continuum. In addition, insufficient vertical confinement allows evanescent coupling of the optical mode into substrate modes, resulting in substrate leakage. In this work, radiation loss is minimized by employing resonators with sufficiently large diameters (3 mm) and a thick thermally grown SiO_2 buffer layer (15 μm) on the substrate. As shown in Fig. 2b of the main

text, numerical simulations indicate that the radiation loss is negligible for the resonator geometries and index contrast (~4%) used here.

Taken together, these analyses establish a clear hierarchy of loss mechanisms: after furnace reflow, scattering and radiation losses are strongly suppressed, and the remaining loss is dominated by absorption-related processes. This conclusion provides a consistent physical framework for interpreting the measured Q values and their dependence on wavelength and waveguide geometry.

2 Details on device characterization and design

2.1 Thickness and refractive index uniformity across the wafer

To assess the Ge uniformity of the film, we performed thickness and refractive index measurements at multiple sites across a 100 mm diameter wafer fabricated with the same composition as those used in this work, but with a slightly thinner layer. As shown in Fig. S4, the film thickness is 8.679 μm on average, with a standard deviation of 0.045 μm (~0.5%). The refractive index at 1550 nm is 1.5152 with a standard deviation of 0.00011 (~0.007%). Measurement uncertainties, determined from repeated measurements at the same site, are 0.012 μm for thickness and 6×10^{-5} for refractive index. These results indicate excellent wafer-scale uniformity.

2.2 Run-to-run stability and long-term process reproducibility

We also evaluated run-to-run repeatability by analyzing depositions over a one-year period. For identical process recipes, the deposition rate is ~0.69 μm per pass. The repeatability uncertainty is 6.82×10^{-3} μm per pass (~1%) for thickness and 8.64×10^{-4} for refractive index, demonstrating strong compositional consistency across runs. Importantly, no evidence of local Ge enrichment or compositional fluctuation was observed within measurement resolution. The small index variation and high Q factors achieved after reflow further suggest that bulk scattering induced by compositional inhomogeneity is negligible.

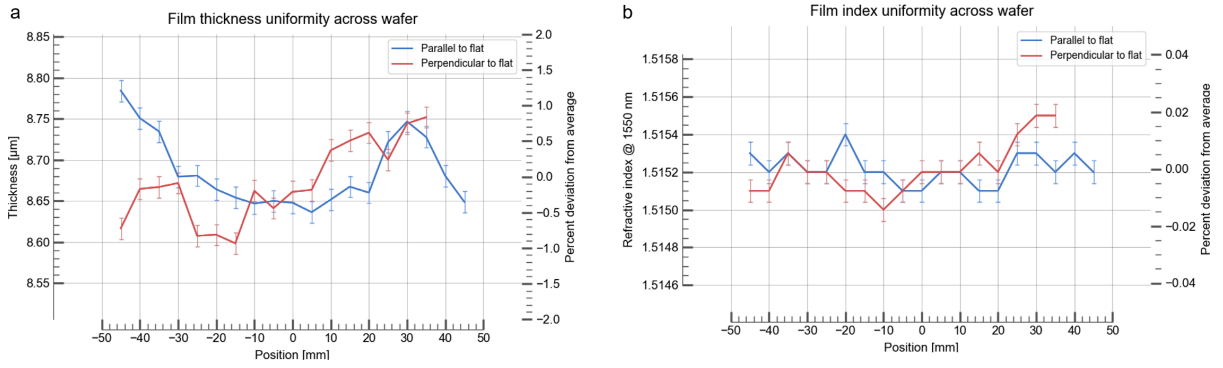

Fig. S4: **Wafer-scale thickness and refractive index uniformity.** Measured film thickness (a) and refractive index at 1550 nm (b) across multiple sites on a single wafer. The average thickness is 8.679 μm with a standard deviation of 0.045 μm (~0.5%), and the average refractive index is 1.5152 with a standard deviation of 0.00011 (~0.007%). Error bars represent measurement uncertainty determined from repeated measurements at the same site.

2.3 Rationale for the GeO_2 doping concentration

The selection of ~50 mol% GeO_2 follows deliberate materials and process considerations beyond its role in increasing refractive-index contrast. At this composition, the germanosilicate glass readily undergoes viscous sintering and reflow above 1250 °C. In contrast, conventional FHD silica processes with lower dopant concentrations rely on boron and/or phosphorus to induce sintering. However, boron is known to induce phase separation and optical loss in fibre-based systems. The higher Ge content in our process eliminates the need for boron, thereby avoiding these drawbacks. Furthermore, processing above the melting point of GeO_2 (1115 °C) ensures the removal of any residual crystallinity originating from the FHD deposition. As for the process window, the accessible GeO_2 compositional range in GeO_2–SiO_2 glasses is relatively broad. Stable glasses from ~40 mol% up to ~90 mol% GeO_2 have been reported, depending on substrate and processing conditions (M. T. Turvey, *Towards micro-ring resonators for quantum sources and rotation sensors*, PhD thesis, University of Southampton (2020)). However, increasing GeO_2 content raises the coefficient of thermal expansion (CTE) and associated thermal stress. For example, 50 mol% GeO_2–SiO_2 glass has a reported CTE of ~4 ppm K^{-1} (Phys. Chem. Glasses Eur. J. Glass Sci. Technol. B **47**, 182–185 (2006)). At higher GeO_2 fractions, cracking and mechanical instability become increasingly

significant, particularly during cooling and furnace reflow. Therefore, 50 mol% GeO_2 represents a deliberate trade-off between index enhancement and mechanical/process stability rather than an empirical choice.

2.4 Waveguide morphology evolution during thermal reflow

Under sufficiently high temperature and annealing time, the etched ridge waveguides undergo viscous reflow driven by surface tension. As illustrated in Fig. S5, the initially rectangular cross-section gradually evolves into a rounded geometry.

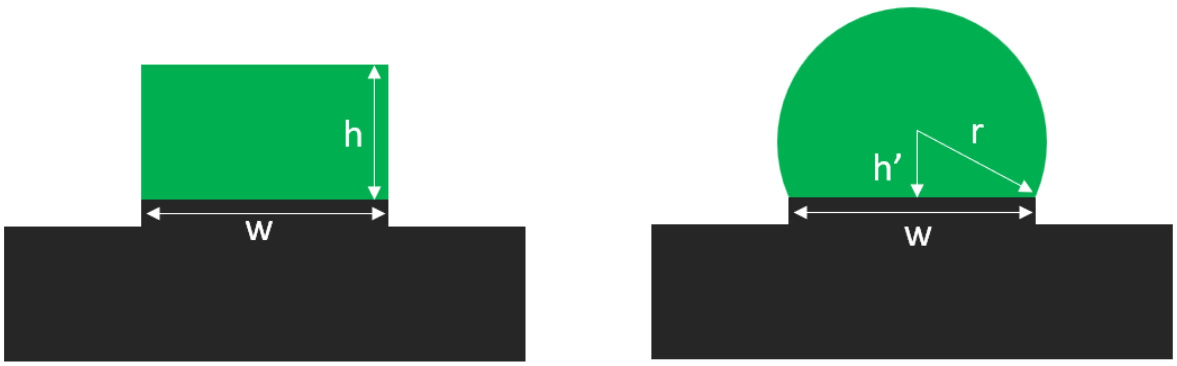

Fig. S5: Schematic illustration of waveguide cross-section evolution during thermal reflow. Left: rectangular ridge prior to reflow. Right: rounded cross-section after reflow, approximated as a truncated circle. The green region represents the Ge:silica core, and the black region denotes the underlying thermal oxide. The drawings are not to scale.

In our process, the underlying thermal oxide is slightly over-etched prior to reflow. Since the thermal oxide consists of pure silica with significantly higher viscosity at the processing temperature, its geometry remains effectively unchanged during annealing. As a result, the bottom interface of the waveguide is preserved, while the Ge:silica core reflows above it.

Because the reflow process is governed by viscous flow without material removal, the total cross-sectional area is conserved. As a simplified geometrical model, we approximate the reflowed cross-section as a truncated circle. Under the constraint of constant area, an initial ridge waveguide with width w and height h is mapped to a truncated circular segment with radius r, whose center

is located at a vertical distance h' from the thermal oxide interface.

$$A = wh = \begin{cases} \pi r^2 \frac{2\pi - 2\arccos(h'/r)}{2\pi} + h'\frac{w}{2}, & h' \geq 0 \\ \pi r^2 \frac{2\arccos(|h'|/r)}{2\pi} - |h'|\frac{w}{2}, & h' < 0 \end{cases} \tag{2}$$

When $h' = 0$, the reflowed geometry reduces to an exact semicircle. For a fixed initial height h, sweeping the initial width w shows that the semicircular condition occurs at

$$w = \frac{8h}{\pi}.$$

In this work, $h = 10\ \mu$m and w ranges from 3 μm to 30 μm.

3 Comparison with Ge:silica and other integrated photonic platforms

3.1 Comparison of Ge:silica integrated photonic platforms

As summarized in Table S1, the present work differs from early FHD PLC platforms in several fundamental aspects:

(1) Ultrahigh-Q regime: We significantly extend device performance into the ultrahigh-Q regime. The demonstrated intrinsic Q of 566 million represents the highest reported value for Ge:silica integrated resonators, and, to our knowledge, this work also provides the first demonstration of visible-wavelength ultrahigh-Q operation in FHD-based Ge:silica platforms, enabling coherence-critical applications beyond the original PLC scope.

(2) High-Ge binary core design: Earlier planar FHD platforms typically sintered silica-rich multicomponent glasses, with Ge primarily introduced for refractive-index tuning. In contrast, the present work deliberately engineers high-Ge binary Ge:silica core glasses, exploiting viscous-flow properties that were historically avoided in FHD processing. This expanded compositional window enables reduced bending radii, enhanced Brillouin gain, lower glass viscosity, and controlled thermal reflow behavior.

(3) Systematic loss and reflow investigation: As described above, this work presents a systematic study of loss mechanisms and establishes furnace reflow as a defect-healing strategy, including

quantitative analysis of scattering suppression, statistical Q distributions, and geometry-dependent performance.

The central achievement of this work is the demonstration that planar flame-hydrolysis-deposited Ge:silica, when combined with post-etch surface-energy-driven thermal reflow, can reliably operate in the ultralow-loss, ultrahigh-coherence regime required for modern microresonator applications. The additional aspects outlined above serve as enabling mechanisms that collectively establish this platform-level advance.

Table S1: **Comparison of Ge:silica integrated photonic platforms.** Early Ge:silica planar lightwave circuits (PLCs) are referenced from [A. Himeno, K. Kato, and T. Miya, "Silica-Based Planar Lightwave Circuits," IEEE J. Sel. Top. Quantum Electron. **4**, 913 (1998)].

Property	This work	Nature 649, 338 (2026)	Early PLC
Deposition method	FHD	PECVD	FHD
Deposition rate	High	Moderate	High
GeO_2 concentration	50%	25%	10–25%
Index contrast	4%	2%	0.45–2%
Bending radius (1550 nm)	~100 μm	~800 μm	~5 mm
Reflow ability	High	Moderate	Low
Highest achievable Q	5.66×10^8	4.6×10^8	4×10^7
Systematic reflow study	Yes	Limited	No
Q statistics and analysis	Yes	Limited	No

3.2 Comparison with other ultralow-loss integrated photonic platforms

Compared with representative Si_3N_4 and $LiNbO_3$ platforms (Table S2), the Ge:silica platform is distinguished by its ultrahigh Q factor, especially at short wavelengths and in large-mode-area geometries, which can reduce thermal noise and improve power handling. These features make it particularly well suited for coherence-critical and high-power applications, such as narrow-linewidth lasers, amplifiers, precision measurements, and quantum photonics. In contrast, Si_3N_4 and $LiNbO_3$ platforms offer higher integration density and stronger nonlinear or electro-optic functionality, but at the expense of higher propagation loss and reduced coherence time. We emphasize that the

Ge:silica platform is not intended to replace these platforms, but rather to complement them by addressing application regimes where loss and coherence are the dominant performance constraints.

Table S2: **Quantitative comparison of representative integrated photonic platforms.** Ge:silica can be deposited via two approaches: flame hydrolysis deposition (FHD, this work) and plasma-enhanced chemical vapor deposition (PECVD; Nature 649, 338–344 (2026)). The corresponding thermal budgets depend on the deposition temperature, approximately 1360 °C for FHD and 270 °C for PECVD. The bending radii listed here are evaluated at 1550 nm for consistent comparison. Notably, Ge:silica can maintain ultrahigh-Q down to the visible regime, where the shorter wavelength enables further reduction of the bending radius.

**Property**	**Ge:silica**	**Si_3N_4**	**$LiNbO_3$**
Refractive index	~1.48–1.54	~2.0	~2.2
Process temperature budget	270 °C (PECVD); 1360 °C (FHD)	~1200 °C	~300 °C
Achievable Q at 1550 nm	$> 10^8$	10^6–10^7	10^6–10^7
Achievable Q at visible	$> 10^8$	10^6	10^6
Bending radius (1550 nm)	~100 μm (air clad)	30–50 μm	20–50 μm
Mode area	20–50 μm^2	1.5 μm^2	1 μm^2
Nonlinear index n_2	3×10^{-20} m^2 W^{-1}	2.5×10^{-20} m^2 W^{-1}	1.8×10^{-20} m^2 W^{-1}
Electro-optic tuning	No	No	Yes
Heterogeneous integration	High	High	Moderate
Application strengths	Loss, coherence-critical systems	Dense nonlinear PICs	EO modulation
